# Study on the Performance of Nano-Titanium Nitride-Coated Stainless Steel Electrodes in Electro-Fenton Systems

**DOI:** 10.3390/nano8070494

**Published:** 2018-07-05

**Authors:** Yita Wang, Youchen Lin

**Affiliations:** Department of Mechanical and Electro-Mechanical Engineering, Ilan University, Yilan City 26047, Taiwan; ytwang@niu.edu.tw; b0022013@ems.niu.edu.tw

**Keywords:** nitriding, nano-TiN, electro-Fenton process, rhodamine B, decolorization

## Abstract

The electro-Fenton (EF) process is a type of electrochemical oxidation process; ·OH radicals are generated on the cathode using electricity and decolorize dye wastewaters. Most studies on EF systems in the past have focused on the operating parameters of this process. In recent years, the influence of electrode performance on the EF process has begun to receive more attention. In this study, direct nitridation was used to prepare titanium nitride powders, which were thereafter coated on an SUS304 stainless steel substrate. The performance of this system in the treatment of rhodamine B dye wastewaters via the EF process was investigated. The experimental methods used in this work include: (1) scanning electron microscopy (SEM); (2) X-ray diffraction (XRD); (3) electrochemical Tafel curves; (4) linear sweep voltammetry (LSV); (5) and cyclic voltammetry (CV). It was shown that high-purity TiN can be formed at nitriding temperatures above 900 °C, and the strength of the (111) crystal plane increases with the increase in nitriding temperature; the TiN coating effectively activates the reactive surface of the electrode owing to its porous structure. In terms of corrosion resistance, the corrosion potential and corrosion current of the TiN 1000 °C/SUS304 electrode were 116.94 mV and 205 nA/cm^2^, respectively, and the coating had a coating porosity of 0.89 × 10^−7^. As compared with SUS304 stainless steel, the TiN 1000 °C/SUS304 composite electrode had a significantly greater degree of corrosion resistance and exhibited higher redox activity in LSV tests. This composite electrode could achieve a decolorization rate of 49.86% after 30 min, and 94.46% after 120 min. In summary, the TiN 1000 °C/SUS304 composite electrode is very stable and has excellent decolorization efficacy in the EF process. Our findings will serve as a useful reference for future studies on EF electrodes.

## 1. Introduction

The environmental hazards associated with industrial pollution and energy production have always been treated as issues of utmost importance by countries around the world. The leakage of industrial wastewaters, contaminated soils, and hazardous organic pollutants to the environment will exacerbate the pollution of water resources [1]. The textile industry in particular consumes over 10,000 tons of dye per annum, and also discharges 100 tons of dye into water systems each year [2]. However, ordinary wastewater treatment techniques cannot process organic dye wastewaters in an effective manner. In recent years, wastewater treatment techniques based on advanced oxidation processes (AOPs) have received increasing attention. An AOP produces hydroxyl radicals (∙OH), which can be used to degrade a target pollutant. The electro-Fenton (EF) process is an especially effective wastewater treatment technique; the reactions of this system consist of three reciprocal chemical reactions that form a self-sustaining chemical system [3]. First, a reduction reaction occurs at the cathode of the EF cell to produce H_2_O_2_, as shown in Equation (1). The Fe^3+^ ions contained in the wastewaters in the cell will be reduced to Fe^2+^ by electrons on the cathodic surface, as shown in Equation (2). The H_2_O_2_ thereafter reacts with Fe^2+^ to undergo the Fenton reaction, thus producing hydroxyl radicals (∙OH), as shown in Equation (3). Finally, the system regenerates itself to perform the electro-Fenton advanced oxidation process (EF-AOP), thus realizing the degradation of pollutants [4].

(1) $$\;O_{2}+2H^{+}+2e^{-}\to H_{2}O_{2}$$

(2) $$\;{\mathrm{Fe}}^{3+}+e^{-}\to {\mathrm{Fe}}^{2+}$$

(3) $$\;H_{2}O_{2}+{\mathrm{Fe}}^{2+}+H^{+}\to {\mathrm{Fe}}^{3+}+H_{2}O+\cdot \mathrm{OH}$$

Numerous factors can affect the degradation performance of EF systems, and the electrode material is one of the most important parameters influencing the efficacy of the EF process. Therefore, the improvement of characteristics of the electrode material is an important subject for studies on the EF process. Cathode materials must simultaneously possess a high level of conductivity, large specific surface areas, high porosities, excellent H_2_O_2_ productivity [5], and high levels of chemical stability [6]. Nidheesh and Gandhimathi [7] noted that the efficiency of EF reactions is determined by cathode properties (the working electrode). Carbon electrodes are highly porous and possess high levels of conductivity and chemical stability; therefore, they are the most frequently used type of electrode in EF studies. Stainless steel also possesses high levels of conductivity and an acceptable level of chemical stability. Chou et al. [8] used stainless steel as an EF electrode for the reduction of Fe^3+^ to Fe^2+^ (similar to Equation (2)) and proved that stainless steel electrodes can outperform graphite electrodes in this particular application. SUS304 stainless steel is the most commonly used type of stainless steel for EF processes. Borràs et al. [9] used SUS304 stainless steel as a cathode in the electrochemical treatment of atrazine and achieved total organic carbon (TOC) removal of 94%. Da Pozzo et al. [10] used AISI 304 stainless steel as a cathode for the treatment of amitrole and achieved TOC removal of 94% after 3 h of treatment. Ramírez et al. [11] used AISI 304 stainless steel as a cathode for the decolorization of methyl orange azo dye wastewaters and achieved a decolorization rate of 94%. However, Rosales et al. [12] noted that stainless steel electrodes tend to gradually corrode during the EF process, which leads to a decline in decolorization rates. Liu and Zhang [5] also observed that EF processes are usually performed in acidic environments; this renders the electrodes susceptible to damage and increases treatment costs. Hence, stainless steel can be used as EF electrodes as they are conductive and amendable to processing, but further improvement is required in terms of corrosion resistance and specific surface area.

Titanium nitride (TiN) has excellent physical and chemical properties, for example, high melting points, high hardness, high corrosion resistance, and high levels of electrical and thermal conductivity [13,14]. Ru et al. [15] noted that TiN coatings can improve the conductivity, corrosion resistance, wear resistance, shear modulus, and creep performance of a material. TiN is currently being used as battery electrodes, capacitor electrodes, biologically inert coatings, medical implants, and hard coatings for machining tools [16,17]. Liu et al. [18] noted that TiN has high levels of electrochemical stability, excellent conductivity, and superb corrosion resistance, and it can be used to improve the activity and durability of carbon nanotubes (CNTs)/Pt catalysts in direct methanol fuel cells. Mani et al. [19] coated SUS316 stainless steel in a TiN monolayer, and this significantly improved the corrosion resistance of bipolar plates in proton exchange membrane fuel cells without reducing its conductivity; this also solved issues related to the formation of passivation films on stainless steel bipolar plates, which increases resistivity and losses in power. Su et al. [20] used TiN as a cathode and performed electrochemical stability tests at pH 1, 7, and 10; it was shown that stable current densities could be maintained at pH 1, 7, and 10. Jin et al. [21] observed that the supply of electrons to TiN electrodes often resulted in the formation of H_2_O_2_ owing to the difficulty of O–O bond cleavage. It is thus shown that TiN electrodes have excellent electrochemical stability when used as cathodes.

Although TiN has demonstrated excellent performance in various engineering applications, investigations on its use in pollutant treatment are scarce. The cathode for the EF system being used in this work must possess high corrosion resistance, excellent conductivity, large reactive surface area, and the ability to produce H_2_O_2_; TiN has the potential to satisfy these requirements. In this work, TiN was coated on SUS304 stainless steel to prepare a composite electrode. We thereafter investigated the performance of this electrode in the treatment of rhodamine B (RhB) wastewaters.

## 2. Materials and Methods

### 2.1. Preparation of TiN Powders

Commercial P25 TiO_2_ powders (TiO_2_ P25, ENONIK, Essen, Germany) were used as a precursor for the preparation of TiN powders. Nitriding was performed for 3 h at 800, 900, or 1000 °C. The atmosphere composition used for the nitriding is pure NH_3_ gas; the powders were stored in a nitrogen atmosphere after nitriding, and thereafter cooled to room temperature. The powders were subsequently removed from the nitrogen chamber.

### 2.2. Preparation of the TiN/SUS304 Electrode

In this study, SUS304 stainless steel (Qunlong Steels, Yilan, Taiwan) was used as substrates (80 mm × 25 mm × 2 mm); the chemical composition of this steel is presented in Table 1. The polishing of the SUS304 stainless steels was performed from 100 grit up to 2500 grit, followed by washing in ethanol (C_2_H_6_O, Nihon Shiyaku, Japan). The SUS304 substrate was thereafter sonicated in acetone (CH_3_COCH_3_, Nihon Shiyaku, Japan) for 15 min and subsequently in distilled water, followed by drying and storage at room temperature.

To prepare the TiN slurry, an appropriate amount of TiN nanopowder was mixed with the polyvinylidene difluoride (–(C_2_H_2_F_2_)*_n_*–, Solef 6020, SOLVAY, Brussels, Belgium, henceforth abbreviated as PVDF) bonding agent at a ratio of TiN:PVDF = 2:1. This mixture was subsequently mixed with methylpyrrolidone (C_5_H_9_NO, Nihon Shiyaku, Tokyo, Japan, henceforth abbreviated as NMP) at room temperature using a hotplate stirrer (SH-301, SUNTEX Co., New Taipei, Taiwan) for 3–4 h. The mixture was thereafter sonicated using an ultrasonicator (POWER SONIC 405, HWASHIN Co., Yeongcheon, Korea) for 15 min.

The TiN/SUS304 electrode was prepared using a scraper and spin coater (SP1, Power Assist Instrument Scientific Co., Taoyuan, Taiwan). The TiN slurry was poured onto the surface of the SUS304 stainless steel substrate, and a scraper was used to coat the specimen with the slurry; the spin coating method was thereafter used to increase the homogeneity of the coating. After the completion of spin coating, the specimen was placed on a flat heater set at 120 °C and dried in a vacuum oven at 120 °C for 2 h, thus completing the preparation of the specimen.

### 2.3. Electrode Characteristics and Performance Analysis

#### 2.3.1. Analysis of Electrode Surface Micromorphology and Characteristics

A scanning electron microscope (SEM, S-4800, Hitachi, Tokyo, Japan) was used to observe the morphologies of the nanopowders and coatings prepared at different temperatures. Qualitative and semiquantitative elemental analyses were performed using an energy-dispersive spectrometer (EDS, QUANTAX Annular XFlash^®^ QUAD FQ5060, Bruker, Middlesex, NJ, USA). X-ray diffraction (XRD, Ultima IV, Rigaku, Tokyo, Japan) was used to analyze the crystalline phases of the nanonitride powders synthesized at different nitriding temperatures, and the products were identified via comparisons with 21-1272, 21-1276, and 38-1420 JCPDS cards [22,23]. The sizes of each crystalline phase were thereafter calculated using the Scherrer equation. The experimental parameters of the XRD experiment on the nitrided powders were as follows: a copper target was used with a scanning range of 20–80° (2*θ*) and a scanning rate of 2°/min.

#### 2.3.2. Electrochemical Analysis

An electrochemical workstation (Zive SP1, WonATech Co., Seoul, Korea) was used to assess the electrochemical characteristics of the TiN/SUS304 composite electrodes made from TiN powders synthesized at different nitriding temperatures. The dimensions of the Pt plate used for the counter electrode (CE) were 40 mm × 20 mm × 0.2 mm, whereas the dimensions of the working electrode (WE) were 80 mm × 25 mm × 2 mm. Saturated Ag/AgCl was used as the reference electrode (RE). Two methods were used in the electrochemical analysis: we plotted the cathodic voltammetric curves of the electrode surfaces obtained during the EF treatment and the H_2_O_2_ productivity was measured in linear sweep voltammetry (LSV) analysis. Cyclic voltammetry (CV) was used to calculate the reactive area of the electrode, based on the methods of Mousset et al. [24] and the Randles-Sevcik equation [25,26]; the simplified form of this equation is shown in Equation (4), which consists of the key factors (*D*, *n*, *C*, *γ*) in the CV test.

(4) $$\;I_{P}=2.69\times 10^{5}\times AD^{0.5}n^{0.5}\gamma^{0.5}C$$

where *I*_p_: peak current; *A*: the reactive surface area of the electrode; *D*: diffusion coefficient of the analytes; *n*: number of electrons participating in the redox reaction; *γ*: scan rate; *C*: concentration.

The corrosion resistance of the modified EF cathode was measured using Tafel curves. The porosity between the coating and substrate (coating porosity) is also an important parameter for corrosion resistance. Matthes et al. [27] proposed an equation for calculating coating porosity, as presented in Equation (5). In this equation, *P* is the coating porosity, *R*_ps_ is the polarization resistance of the uncoated substrate, *R*_p_ is the polarization resistance of the coated substrate, *∆E*_corr_ is the difference between the corrosion potentials of the coated and uncoated materials, and *β*_α_ is the anodic Tafel slope of the substrate.

(5) $$\;P=\left(\frac{R_{\mathrm{ps}}}{R_{p}}\right)\times 10^{-\left(\Delta E_{\mathrm{corr}}/\beta_{a}\right)}$$

#### 2.3.3. Tests on Rhodamine B (RhB) Decolorization Performance

The tank of the EF system was filled with a solution of RhB xanthene dye (C_28_H_31_C_l_N_2_O_3_, Aldrich, Louis, MO, USA) to simulate the treatment of wastewaters. During the experiment, the wastewaters were aerated at a rate of 50 (mL/min, *T* = 273 K, *P* = 760 torr) and stirred using a magnetic stirrer at 700 rpm. First, the EF reaction was examined after a short time (30 min), and the state of RhB pigment decolorization was observed. Subsequently, the reaction time of the TiN/SUS304 electrode that exhibited the best decolorization performance in the short experiment was extended to 2 h to investigate the stability of the decolorization performance of the electrode. The equation used for calculating the decolorization rate is shown in Equation (6); *C*_0_ is the initial concentration of RhB, *C* is the concentration of RhB after the reaction.

(6) $$\mathrm{Decolorization}\;\mathrm{rate}\;(\mathrm{DC})\;=\frac{\left(C_{0}-C_{t}\right)}{C_{0}}\times 100(\%)$$

The decolorization of RhB in the modified electrode has been described by kinetic fits as shown in Equation (7), where *C* is the chromaticity of concentration of RhB, *m* is the reaction order, *t* is the reaction time, and *k* is the reaction constant.

(7) $$\;\frac{dC}{dt}=-kC^{m}$$

As the *m* is used as 1, the decolorization of RhB in the modified electrode by first-order kinetic fits could be determined by Equation (8), as follows:

(8) $$\;\ln\left(\frac{C_{t}}{C_{0}}\right)=-kt$$

## 3. Results and Discussion

### 3.1. Analysis of the TiN Morphologies and Compositions Corresponding to Different Nitriding Temperatures

#### 3.1.1. Morphological and Compositional Analyses of TiN Powders Formed at Different Nitriding Temperatures

The nitriding temperature determines the degree of TiO_2_ nitrification and thus the formation of TiN. The SEM images correspond to the TiO_2_ powders after nitriding at (a) 800, (b) 900, and (c) 1000 °C. It is shown that the spherical shape of TiO_2_ powders is retained at all nitriding temperatures. Zhong et al. [28] observed that ammonia reduction is a highly effective method for maintaining precursor morphologies. Table 2 presents the results of EDS analyses of the TiO_2_ powders nitrided at different nitriding temperatures. It is shown that the TiO_2_ powders nitrided at 800 °C only present Ti and O_2_ signals; hence, a nitriding temperature of 800 °C is insufficient for converting TiO_2_ into TiN. In contrast, the powders nitrided at 900 and 1000 °C do not present O_2_ signals. In the study by Gou et al. [16], TiO_2_ was used as a precursor for the synthesis of TiN powders via gaseous nitriding; TiN was successfully synthesized at 900 and 1000 °C. Therefore, one may deduce that the TiO_2_ powders nitrided at 900 and 1000 °C in this experiment have been converted into TiN powders.

**Figure 1:**
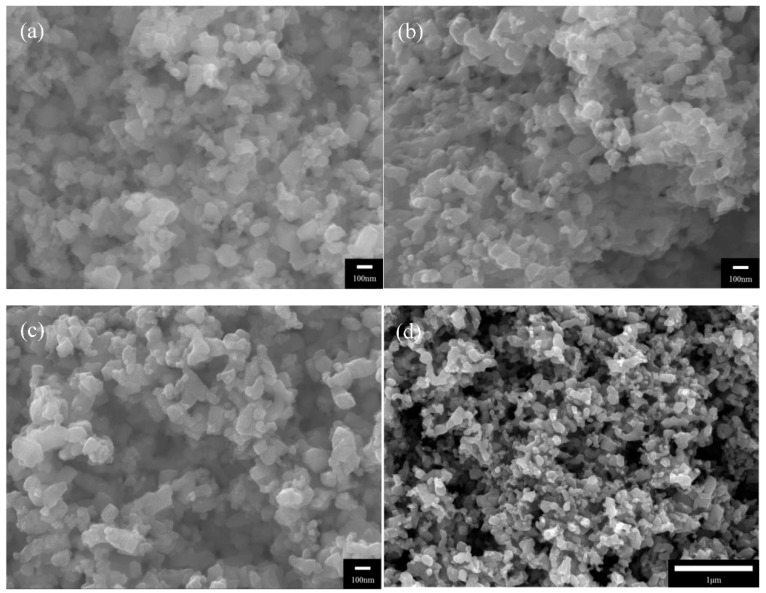
Scanning electron microscopy (SEM) images of the nitrided TiO_2_ powders and the surface of the coating: (**a**) TiO_2_ powder nitrided at 800 °C; (**b**) TiO_2_ powder nitrided at 900 °C; (**c**) TiO_2_ powder nitrided at 1000 °C; (**d**) surface morphology of the TiN coating.

Figure 1d shows an SEM image of the TiN:PVDF = 2:1 coating, which provides a detailed illustration of its micromorphology. It is shown that the coating has a highly porous three-dimensional surface structure and is thus highly viable for application as an EF cathode. Liang et al. [6] noted that cathode conductivity, porosity, and surface electroactivity are crucial parameters for the generation of H_2_O_2_ during the EF process.

It is shown that TiO_2_ powders nitrided at 800, 900, and 1000 °C retain the spherical shape of the TiO_2_ precursor and have a particle diameter of ~100 nm. The coating of nanoscale TiO_2_ on an electrode imbues its surface with a large specific surface area, which is beneficial for increasing the area of contact between the electrode and pollutants in the solution. This will subsequently increase the treatment efficacy of the system. As oxygen was not observed in powders nitrided at ≥900 °C, we have preliminarily identified these powders as TiN powders. Nonetheless, the chemical composition of these powders requires further verification.

#### 3.1.2. Analysis of TiN Powders Prepared at Different Nitriding Temperatures via X-ray Diffraction

**Figure 2:**
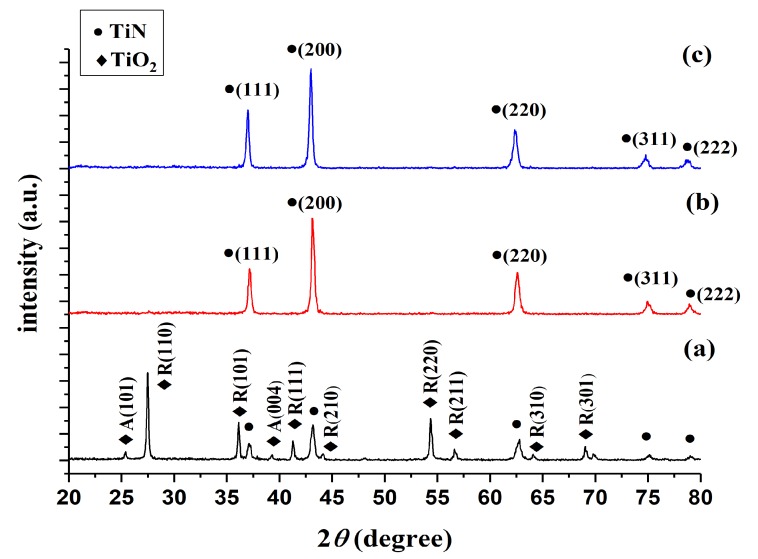
X-ray diffraction (XRD) graphs of TiN powders formed at different nitriding temperatures. Note: A is the anatase phase, whereas R is the rutile phase.

XRD was used to compare the products of TiO_2_ nitriding at 800, 900, and 1000 °C (henceforth referred to as TiN 800 °C, TiN 900 °C, and TiN 1000 °C, respectively) with the 21-1272, 21-1276, and 38-1420 JCPDS cards [22,23], in order to validate the chemical composition of these products. Figure 2 illustrates the XRD graphs of the TiN powders formed at different temperatures; the circular markers (●) indicate TiN peaks, whereas the diamond-shaped markers (◆) indicate TiO_2_ peaks. Figure 2a shows that the TiO_2_ powders nitrided at 800 °C mainly exhibit the characteristic peaks of TiO_2_. As the TiN phase is only weakly crystalline in these powders, its characteristics peaks are relatively weak in the XRD graph. Wetchakun and Phanichphant [29] have shown that the anatase phase of TiO_2_ will transform into the rutile phase when TiO_2_ is subjected to temperatures between 700 and 800 °C; this is consistent with the findings of this experiment. In other words, TiN cannot be fully formed at 800 °C. Dolat et al. [30,31] observed that the modification of TiO_2_ at 800 °C in an ammonia atmosphere produces a nitrogen-doped TiO_2_–TiN composite. Therefore, TiN 800 °C is denoted as TiO_2_ & TiN 800 °C in this study. Figure 2b shows that TiO_2_ powders nitrided at 900 °C no longer exhibit TiO_2_ peaks and only display TiN peaks; hence, we have experimentally demonstrated that TiO_2_ may be converted into TiN via nitriding at 900 °C. Figure 2c shows that the nitriding of TiO_2_ at 1000 °C produces the same results as nitriding at 900 °C, as no TiO_2_ peaks can be observed in this graph. However, as TiN 1000 °C has a greater degree of intensity than TiN 900 °C in the (111) crystal lattice, one may infer that an increase in temperature will promote the growth of crystallinity in the (111) direction. The Scherrer equation was used to calculate the crystal sizes of the (111), (200), and (220) characteristic peaks of TiN 900 and TiN 1000 °C; it was observed that the crystal sizes corresponding to these peaks are 24, 23, and 19 nm in TiN 900 °C, whereas the crystal sizes corresponding to these peaks are 27, 24, and 19 nm in TiN 1000 °C. Hence, an increase in temperature resulted in an increase in the crystal sizes of the (111) and (200) crystal planes. In particular, the (111) crystal plane increased in size by 3 nm, but no significant growth was observed in the (220) plane. These results indicate that the TiN phase cannot be fully formed at nitriding temperatures below 900 °C, whereas TiO_2_ can be nitrided into TiN at temperatures of 900 °C and above. Furthermore, the strength of the TiN (111) crystal plane will increase with the increase in temperature. Delblanc et al. [32] noted that (111)-oriented TiN films display excellent corrosion properties in general, as they have high corrosion potentials and low corrosion current densities. Here, we have determined that the strength of the (111) crystal plane differs in TiN 900 and TiN 1000 °C.

In summary, nitriding temperatures of 900 °C and above can be used to form high-purity TiN. The products formed at different nitriding temperatures are denoted as TiO_2_ & TiN 800, TiN 900, and TiN 1000 °C. TiN 900 and TiN 1000 °C in particular are both high-purity TiN powders, but exhibit differences in the strength of their (111) crystal planes. Hence, the effects of nitriding temperature on corrosion resistance will require further investigation.

### 3.2. Electrochemical Properties of TiN/SUS304

#### 3.2.1. Tafel Corrosion Tests

In most cases, dye wastewaters in the EF reactor are acidic and have pH values of approximately 2–3. The use of metal-based electrodes to perform the EF reaction in acidic solutions tends to lead to electrode corrosion, thus inducing losses in electrode quality and degradations in electrode stability. This ultimately results in significant losses of efficacy in the treatment of wastewaters using the EF process. Therefore, EF electrodes must be highly resistant to corrosion. It is widely accepted that TiN is a ceramic material that is highly resistant to corrosion and is very chemically stable. We have successfully synthesized TiN nanopowders at 900 and 1000 °C; here, the corrosion resistances of these nanopowders were compared in a pH = 3 environment. In addition, SUS304 stainless steel substrates were coated using TiN, which serve as a corrosion-resistant coating, and Tafel curve analyses were performed on these coated electrodes. The corrosion resistances of SUS304 stainless steels with TiN coatings synthesized at different temperatures were thus compared in an acidic environment (pH = 3).

**Figure 3:**
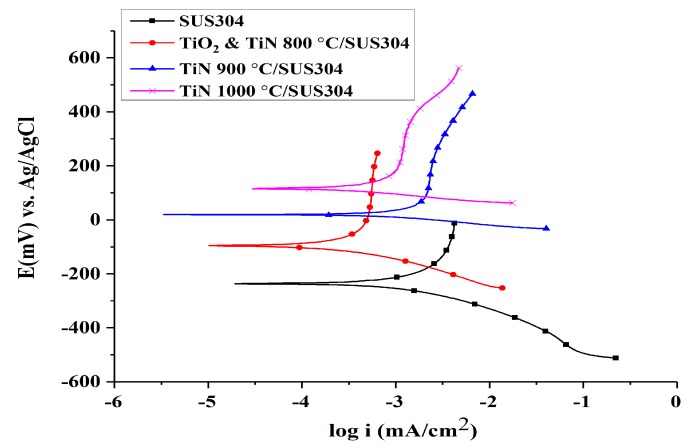
Tafel curves for coated and uncoated SUS304 cathodes in an EF system. Note: The solution contains 0.1 M KNO_3_ and 20 ppm of Fe^2+^. The pH value of the solution was adjusted to pH = 3 using 1 M HNO_3_.

Figure 3 illustrates the Tafel curves of SUS304 with different coatings, whereas Table 3 compares the Tafel properties of SUS304 with different coatings. It is shown that TiN 1000 °C has the highest corrosion potential, followed by TiN 900 °C/SUS304, TiO_2_ & TiN 800 °C/SUS304, and finally SUS304; the corresponding corrosion potentials are 116.94, 20.016, −96.522, and −233.607 mV, respectively. The corrosion resistance of a material in an environment is proportional to its corrosion potential; hence, TiN 1000 °C/SUS304 has the best corrosion resistance between these electrodes. The corrosion current of TiN 900 °C/SUS304 is the highest of these electrodes, followed by those of SUS304, TiN 1000 °C/SUS304, and finally TiO_2_ & TiN 800 °C/SUS304; the corresponding currents are 524, 325, 205, and 85.8 nA/cm^2^, respectively. The corrosion current indicates the rate of corrosion after a material has begun to corrode; the greater the corrosion current, the greater the rate of corrosion. Therefore, TiO_2_ & TiN 800 °C/SUS304 has the slowest rate of corrosion as it has the lowest corrosion current, and TiN 900 °C/SUS304 has a greater corrosion current than SUS304. Wang et al. [33] observed that defects in TiN coatings could induce greater corrosion currents in coated materials than those of the substrate, in addition to galvanic corrosion between the coating and substrate. This further accelerates the substrate solvation and gradually increases the corrosion current densities over time. The coating porosities of TiO_2_ & TiN 800 °C/SUS304, TiN 900 °C/SUS304, and TiN 1000 °C/SUS304 were calculated using Equation (5), and they were determined to be P 0.1084 × 10^−4^, P 0.92 × 10^−4^, and P 0.89 × 10^−7^, respectively. Thus, the coating porosities of TiO_2_ & TiN 800 °C/SUS304 and TiN 900 °C/SUS304 are three orders of magnitude greater than that of TiN 1000 °C/SUS304. Valdez et al. [34] noted that coating porosity is closely related to the quality of the coating and has a direct impact on the corrosion resistance of the coating. The presence of gaps and pinholes in a coating could reduce its density to levels below that of the substrate, and low porosities could prevent the passage of corrosive solutions into the substrate through the coating, thus weakening local corrosion kinetics.

In summary, the TiO_2_ & TiN 800 °C/SUS304 electrode has the lowest corrosion current, but has a lower corrosion potential than TiN 1000 °C/SUS304. In addition, the coating porosity of TiN 1000 °C/SUS304 is lower than those of TiO_2_ & TiN 800 °C/SUS304 and TiN 900 °C/SUS304 by three orders of magnitude. Therefore, TiN 1000 °C/SUS304 has the highest corrosion potential, the second lowest corrosion current, and the lowest coating porosity. Hence, TiN 1000 °C/SUS304 has the highest resistance to corrosion. Rosales et al. [12] observed that electrode corrosion reduces the decolorization rate of stainless steel electrodes in the EF reaction. Therefore, the coating of TiN 1000 °C on a SUS304 substrate may be expected to resolve the corrosion issues afflicting stainless steel electrodes and maintain electrode stability during wastewater treatments.

#### 3.2.2. Linear Sweep Voltammetry (LSV)

**Figure 4:**
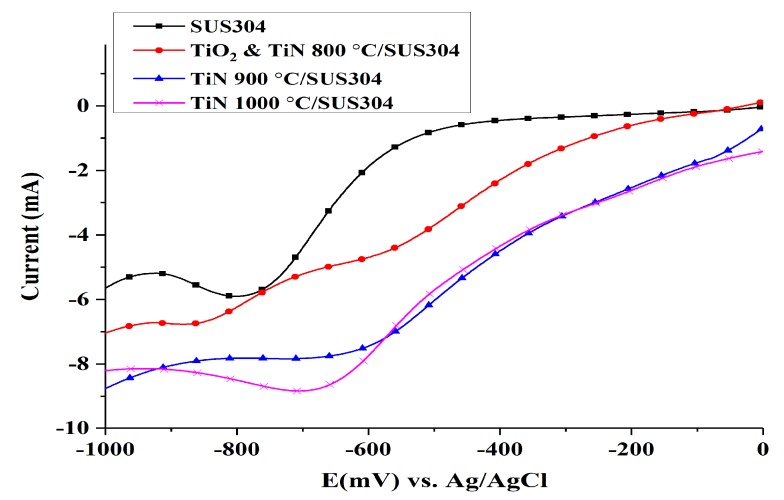
Linear sweep voltammograms of coated and uncoated SUS304 cathodes in the electro-Fenton (EF) system. Note: 0.1 M KNO_3_ solution was used, and 1 M HNO_3_ was used to adjust the pH of the solution to pH = 3. O_2_ aeration was performed for 10 min, and the sweep rate was 10 mV/s.

LSV was used to measure the oxygen reduction reaction (ORR) and to investigate the efficacy of the coated SUS304 electrode in terms of H_2_O_2_ production. The results of the LSV tests are shown in Figure 4. SUS304 exhibits its highest response current at a potential of −800 mV, which indicates that ORR occurs at this potential to produce H_2_O_2_; at −900 mV, the current begins to decrease owing to the production of H_2_ and H_2_O. The LSV curve of TiO_2_ & TiN 800 °C shows that a constant current plateau is formed between −800 and −950 mV; this reaction interval should correspond to the limiting current region [35]. The current begins to increase after the potential exceeds −950 mV, which indicates the participation of the hydrogen evolution reaction. The linear voltammetry curve of TiN 900 °C/SUS304 shows a linear decrease in response current in the 0 to −600 mV range. A plateau appears between −600 and 850 mV, which indicates that the response current is constant within this range of potentials. Yang et al. [35] noted that this plateau corresponds to the limiting current region, where H_2_O_2_ is produced. The production of H_2_O_2_ occurs via mass transfers of dissolved oxygen through the cathode solution diffusion layer, rather than electron transfers between dissolved oxygen and the cathode. Therefore, even with a further increase in potential, the cathode cannot directly react with dissolved oxygen. At potentials between −900 and −1000 mV, the interaction between H_2_ and H_2_O production reduces the response current. The linear voltammetry curve of TiN 1000 °C/SUS304 indicates that its response curve from the initial potential to −600 mV largely overlaps with that of TiN 900 °C/SUS304. However, the response current of TiN 1000 °C/SUS304 continues to increase with the increase in potential, and reaches its maximum at −700 mV. A further increase in potential leads to a gradual decline in the response current of TiN 1000 °C/SUS304; the changes in its response current become more gradual after −900 mV. It is shown that the optimal range of potentials for the production of H_2_O_2_ is −600 to −900 mV. The response current does not increase when the potential increases to 1000 mV; therefore, the TiN 1000 °C/SUS304 electrode can effectively delay the appearance of the hydrogen evolution potential.

In summary, the TiO_2_ & TiN 800 °C/SUS304, TiN 900 °C/SUS304, and TiN 1000 °C/SUS304 coated electrodes generally increase reduction currents relative to SUS304, and may thus be expected to increase the production of H_2_O_2_ [36]. In addition, the optimal working potential for the production of H_2_O_2_ via the reduction reaction is −800 mV in SUS304 stainless steel cathodes. This working potential was thereafter used to perform LSV on TiN-coated electrodes; it was observed that TiN 1000 °C/SUS304 produces the highest response current, followed by TiN 900 °C/SUS304, TiO_2_ & TiN 800 °C/SUS304, and SUS304. The corresponding response current values are −8.5, −8, −6.5, and −6 mA, respectively. It is thus shown that the TiN 1000 °C/SUS304 electrode has the best EF performance among these electrodes. This result was subsequently validated via EF decolorization experiments.

#### 3.3.3. Cyclic Voltammetry Tests

**Figure 5:**
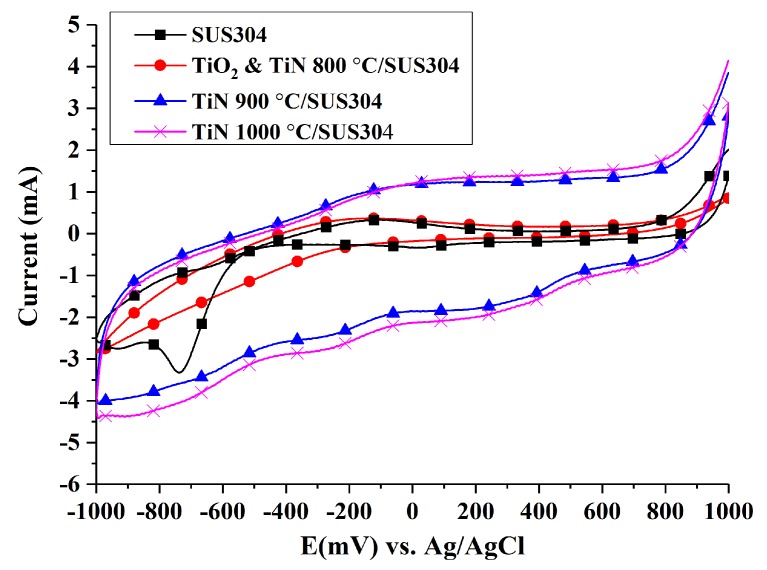
Comparison between cyclic voltammograms of coated and uncoated SUS304 cathodes in the EF system. Note: The solution contains 0.1 M KNO_3_ and 20 ppm Fe^2+^, and its pH value was adjusted to pH = 3 using 1 M HNO_3_.

The reducing power of Fe ions in the system is an important factor in the EF process. Hence, the EF reaction rate is closely related to the rate of Fe^3+^ reduction to Fe^2+^. Figure 5 illustrates the cathodic cyclic voltammograms of coated SUS304 electrodes. An electrochemistry program was performed by the electrochemical workstation (Zive SP1)equipped with IVMAN calculations software, and it was determined that the peak currents (*i*_p_) of each electrode in decreasing order are: TiN 1000 °C/SUS304 (−2.78 mA), TiN 900 °C/SUS304 (−2.45 mA), SUS304 (−1.276 mA), and TiO_2_ & TiN 800 °C/SUS304 (−1.08 mA). The Randles–Sevcik equation (Equation (4)) was used to calculate the reactive surface area of the electrodes, A, through the substitution of peak current values (*i*_p_) (where *D* is 7.60 × 10^−6^ cm^2^/s, *n* = 1, *γ* = 0.01 V, and *C* = 1 × 10^−5^ mol/cm). The results of this calculation are as follows: TiN 1000 °C/SUS304 (3.75 cm^2^), TiN 900 °C/SUS304 (3.3 cm^2^), SUS304 (1.72 cm^2^), and TiO_2_ & TiN 800 °C/SUS304 (1.45 cm^2^). Liang et al. [6] observed that large electroactive surface area and porosity improve the H_2_O_2_ productivity of a cathode. Sirés et al. [37] further noted that the use of highly porous carbon cathodes strengthens the reduction of O_2_ to H_2_O_2_ (Equation (1)) and the reducing power of Fe ions (Equation (2)). This highlights the importance of the increase in electroactive surface area for the EF process, and one may deduce that an increase in the electroactive surface area will accelerate the EF reactions. Additionally, the corrosion current densities calculated by the reactive surface area of each electrode were shown in Table 4. It also indicated that the specimens of TiN 1000 °C/SUS304 had the slowest rate of corrosion.

The high electroactive surface areas of TiN/SUS304 electrodes may be explained by the highly porous 3D surface structure of TiN, which is shown in Figure 1d. This structure is also beneficial for achieving high decolorization rates in EF decolorization tests. TiO_2_ & TiN 800 °C/SUS304 exhibits a relatively small electroactive surface area. This is due to the presence of TiO_2_ in the TiO_2_ & TiN 800 °C/SUS304 coating; TiO_2_ has a lower conductivity than TiN and its presence will interrupt electron transmission pathways, thus reducing the electroactivity in EF system. In summary, TiN 1000 °C/SUS304 and TiN 900 °C/SUS304 have large electrochemically active surface areas, whereas TiN 1000 °C/SUS304 has the largest electrochemically active surface area. Therefore, TiN 1000 °C/SUS304 is the best electrode for EF applications.

#### 3.3.4. Electro-Fenton RhB Decolorization Experiment

The effects of nitriding temperature on the redox power and chemical stability of the coatings have been discussed in previous sections, and it was shown that TiN 1000 °C/SUS304 has the best corrosion resistance and H_2_O_2_ productivity, followed by TiN 900 °C/SUS304. A high degree of corrosion resistance allows an electrode to operate in a stable manner amidst the reactions of the EF system, whereas high redox powers will increase the efficacy of the EF system. Hence, the TiN 1000 °C/SUS304 electrode should exhibit the best performance in wastewater treatment.

**Figure 6:**
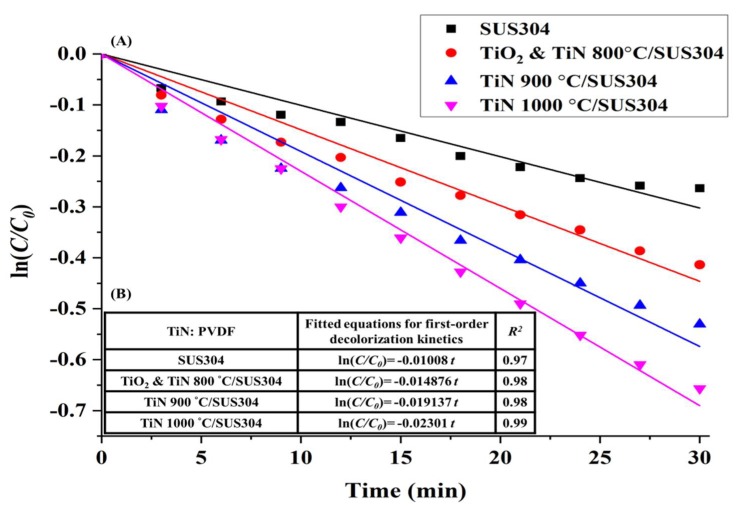
(**A**) First-order kinetic fits of the decolorization process by coated and uncoated SUS304 electrodes in the EF system; (**B**) Fitted equations for the first-order decolorization kinetics of the coated and uncoated SUS304 electrodes in the EF system. Note: The solution contains 0.1 M KNO_3_, 20 ppm of Fe^2+^, and 5 ppm RhB. The working potential was −800 mV, and 1 M HNO_3_ was used to adjust the pH of the solution to pH = 3.

**Figure 7:**
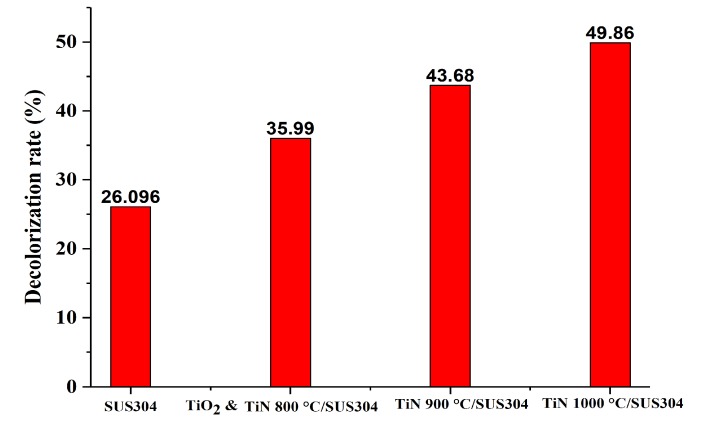
Comparison between the decolorization rates of coated and uncoated SUS304 electrodes in the EF system after 30 min of reaction time.

To validate this hypothesis, a short 30-min-long EF decolorization experiment was first performed. The natural log of the decolorization ratios (*C*/*C*_0_) measured in this experiment was used to perform first-order kinetic equation calculations. The results of these calculations are shown in Figure 6A, which illustrate the first-order kinetic fits for decolorization using coated SUS304 electrodes. Here, it is shown that decolorization efficacy is positively correlated with the nitriding temperature of the coating. This is because an increase in nitriding temperature leads to higher TiN purities and higher levels of crystallinity, which subsequently improves the electrochemical efficacy of the coating. Based on the calculations of Equation (6), it may be inferred that all the ceramic coatings exhibit first-order kinetics in the decolorization reaction, as presented in Figure 6B. The *t* value is time, and the slopes are the decolorization rates. As all the *R*^2^ values are greater than 0.95, it is proven that the coatings uniformly exhibit first-order kinetics in the decolorization reaction. It is presented in Figure 6B that the slopes of SUS304, TiO_2_ & TiN 800 °C, TiN 900 °C/SUS304, and TiN 1000 °C/SUS304 are −0.01008, −0.014876, −0.019137, and −0.02301, respectively. As TiN 1000 °C/SUS304 has the steepest slope, it has the best decolorization rate per unit time. Figure 7 illustrates the decolorization rates of the coated and uncoated SUS304 electrodes in the EF system after 30 min. The decolorization rates in decreasing order are TiN 1000 °C/SUS304, TiN 900 °C/SUS304, TiO_2_ & TiN 800°C/SUS304, and SUS304, and their decolorization rates are 49.86%, 43.68%, 35.99%, and 23.096%, respectively. As compared with the uncoated SUS304 stainless steel electrode, the composite coatings effectively increase the decolorization efficacy by factors between 1.38 and 1.91. The commendable performance of these coatings is further validated by the results of the LSV experiment, which demonstrate that the TiN coatings possess high levels of redox power. Wang et al. [36] also noted that reducing currents are positively correlated with the treatment efficacy; furthermore, the nanopowders provide a large electroactive area of contact and high porosities [6]. The TiN 1000 °C/SUS304 electrode also exhibits a 6.18% increase in decolorization rates over the TiN 900 °C/SUS304 electrode; as it was shown in the LSV experiments that the response current corresponding to the reduction peak was 0.5 mA higher in the former, the increase in H_2_O_2_ productivity is correlated with the response current. The short EF experiment demonstrates that TiN coatings have high decolorization rates, and the TiN 1000 °C/SUS304 electrode has the best performance in wastewater treatment. A two-hour-long EF experiment was thereafter performed using the TiN 1000 °C/SUS304 electrode to investigate the stability of its performance in the EF reaction in detail.

**Figure 8:**
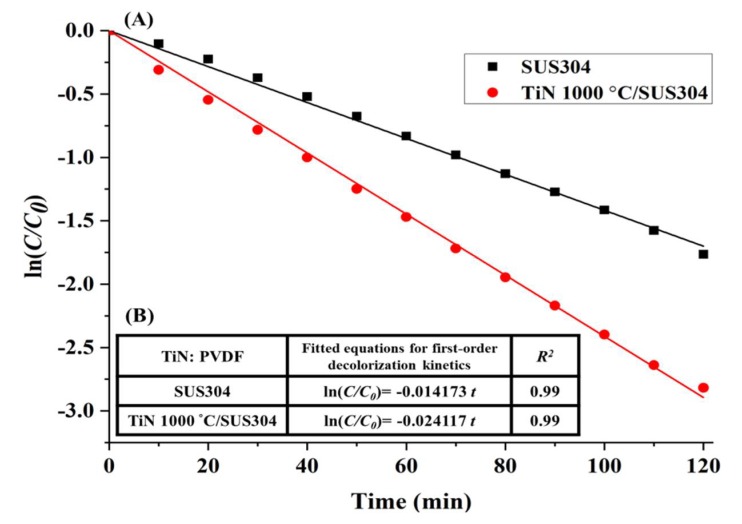
(**A**) First-order decolorization kinetics of the TiN 1000 °C/SUS304 electrode in the EF system; (**B**) Fitted equation for the first-order decolorization kinetics of the TiN 1000 °C/SUS304 electrode. Note: The solution contains 0.1 M KNO_3_, 20 ppm of Fe^2+^, and 5 ppm of RhB. The working potential was −800 mV, and the pH of the solution was adjusted to pH = 3 using 1 M HNO_3_.

The TiN 1000 °C/SUS304 electrode exhibits first-order decolorization kinetics over 2 h of reaction time, as shown in Figure 8A. The first-order kinetic equations of the decolorization reactions are presented in Figure 8B; as the R^2^ values are all greater than 0.95, all the electrodes exhibit first-order decolorization kinetics. In particular, the decolorization rate of TiN 1000 °C/SUS304 after 120 min was −0.024117, which is similar to its decolorization rate in Figure 6B. Hence, the TiN 1000 °C/SUS304 electrode is stable in the electrochemical treatment environment throughout the longer experiment. This also proves that the decrease in decolorization rates caused by corrosion of the stainless steel electrode, as mentioned by Rosales et al. [12], can be prevented using TiN coatings. The decolorization rates of TiN 1000 °C/SUS304 and SUS304 after 120 min of decolorization are 94.46% and 81.74%, respectively. This demonstrates the superior performance of the TiN 1000 °C-coated SUS304 electrode in the decolorization process.

In summary, the TiN 1000 °C/SUS304 electrode is highly resistant to corrosion and exhibits the best decolorization performance in the EF treatment of RhB wastewaters. The coating of TiN 1000 °C also allows the SUS304 electrode to maintain a stable level of performance over longer timescales. Furthermore, the high redox powers and large electroactive surface area of the TiN coating also increase the efficacy of the EF process.

## 4. Conclusions

In this study, we have investigated the performance of nano-titanium nitride-coated stainless steel cathodes in the treatment of RhB wastewaters via the EF process. Our findings are as follows:

1. The direct nitriding method was used to prepare TiN nanopowders. The powder formed at a nitriding temperature of 800 °C was a composite nanopowder containing TiO_2_ and TiN; TiO_2_ exhibited anatase crystal orientations in the (101) direction of 2*θ* = 26° and (004) direction of 2*θ* = 36°, whereas it exhibited rutile crystal peaks in all other directions.
2. The nanopowders nitrided at different nitriding temperatures were used to coat electrodes, and it was observed that the TiN 1000 °C coating exhibited the best performance in electrochemical performance tests. In the Tafel test, it was shown that the corrosion potential and corrosion current of TiN 1000 °C were 116.94 mV and 205 nA/cm^2^, respectively. Based on the LSV tests, the optimal range of potentials for the production of H_2_O_2_ was −600 to −900 mV, and the potential of −800 mV produced the optimal response current (−8.5 mA). Cyclic voltammetry tests indicated that TiN 1000 °C has the highest electrochemically active surface area at 3.75 cm^2^; hence, the TiN 1000 °C coating can be used to increase the rate of EF reaction.
3. The EF decolorization performance of the electrodes coated using nanopowders nitrided at different temperatures indicate that the decolorization performance is positively correlated with TiN crystallinity. The TiN 1000 °C/SUS304 electrode exhibited the best performance in EF decolorization. After 30 min of reaction time, the decolorization rate of TiN 1000 °C/SUS304 was 1.91 times that of the SUS304 substrate. In the two-hour-long decolorization experiment, it shown that the first-order kinetics of TiN 1000 °C/SUS304 exhibited the same slope in the short and long experiments, which demonstrates that the TiN 1000 °C coating possesses a high degree of electrochemical stability.

In this study, an SUS304 stainless steel substrate was coated with a nano-TiN transition metal nitride coating to replace conventional mechanically processed 3D carbon materials in EF systems. It was shown that this composite electrode exhibits excellent decolorization efficacies for RhB wastewaters. We have thus provided a pathway for improving electrode performance in electrochemical wastewater treatments and designed a type of electrode with significant potential for application.

## Figures and Tables

**Table 1:** Chemical composition of SUS304 stainless steel.

Chemical Composition of SUS304 Stainless Steel										
**Chemical Components**	**C**	**Si**	**Mn**	**P**	**S**	**Ni**	**Cr**	**N**	**Mo**	**Cu**
**Contents (%)**	0.042	0.47	0.97	0.029	0.004	8.09	18.06	0.058	0.00	0.02

**Table 2:** Results of energy-dispersive spectrometer (EDS) analysis of the TiO_2_ powders nitrided at different temperatures.

	Element	O	N	Ti
Powder				
800 °C		76.31	-	Bal.
900 °C		-	48.32	Bal.
1000 °C		-	49.42	Bal.

Bal.: The balance for percentage of chemical composition.

**Table 3:** Tafel properties of coated and uncoated SUS304 electrodes in an EF system.

	Properties	Ecorr (mV)	Icorr (nA/cm^2^)	Rp (kΩ/cm^2^)	P (10^−4^)
Materials					
**SUS304**		−233.607	325	28.093	-
**TiO_2_ & TiN 800** **°**C**/SUS304**		−96.522	85.8	114.65	0.1084
**TiN 900** **°**C**/SUS304**		20.016	524	16.46	0.92
**TiN 1000** **°**C**/SUS304**		116.94	205	41.367	0.89 × 10^−3^

**Table 4:** Corrosion current of the reactive surface area of coated and uncoated SUS304 electrodes in an electro-Fenton (EF) system.

Materials	Icorr (nA)	Reactive Surface Area (cm^2^)	Icorr (nA/cm^2^)
**SUS304**	14,365	1.72	8351.744
**TiO_2_ & TiN 800 °C/SUS304**	3792.36	1.45	2615.421
**TiN 900 °C/SUS304**	23,160.8	3.3	7018.424
**TiN 1000 °C/SUS304**	9061	3.75	2416.267

## References

[1] Babuponnusami A., Muthukumar K. (2014). A review on Fenton and improvements to the Fenton process for wastewater treatment. J. Environ. Chem. Eng..

[2] Yagub M.T., Sen T.K., Ang H.M. (2012). Equilibrium, Kinetics, and Thermodynamics of Methylene Blue Adsorption by Pine Tree Leaves. Water Air Soil Pollut..

[3] Qiang Z.M. (2002). Removal of Selected Hazardous Organic Compounds by Electro-Fenton Oxidation Process. Ph.D. Thesis.

[4] Garcia-Segura S., Centellas F., Arias C., Garrido J.A., Rodriguez R.M., Cabot P.L., Brillas E. (2011). Comparative decolorization of monoazo, diazo and triazo dyes by electro-Fenton process. Desalination.

[5] Liu W., Ai Z., Zhang L. (2012). Design of a neutral three-dimensional electro-Fenton system with foam nickel as particle electrodes for wastewater treatment. J. Hazard. Mater..

[6] Liang P., Rivallin M., Cerneaux S., Lacour S., Petit E., Cretin M. (2016). Coupling cathodic Electro-Fenton reaction to membrane filtration for AO7 dye degradation: A successful feasibility study. J. Membr. Sci..

[7] Nidheesh P.V., Gandhimathi R. (2012). Trends in electro-Fenton process for water and wastewater treatment: An overview. Desalination.

[8] Chou S., Huang Y.H., Lee S.N., Huang G.H., Huang C. (1999). Treatment of high strength hexamine-containing wastewater by electro-Fenton method. Water Res..

[9] Borràs N., Oliver R., Arias C., Brillas E. (2010). Degradation of Atrazine by Electrochemical Advanced Oxidation Processes Using a Boron-Doped Diamond Anode. J. Phys. Chem. A.

[10] Pozzo A.D., Merli C., Sirés I., Garrido J.A., Rodríguez R.M., Brillas E. (2005). Removal of the herbicide amitrole from water by anodic oxidation and electro-Fenton. Environ. Chem. Lett..

[11] Ramírez C., Saldaña A., Hernández B., Acero R., Guerra R., Segura S.G., Brillas E., Hernández J.M.P. (2013). Electrochemical oxidation of methyl orange azo dye at pilot flow plant using BDD technology. J. Ind. Eng. Chem..

[12] Rosales E., Pazos M., Longo M.A., Sanromán M.A. (2009). Electro-Fenton decoloration of dyes in a continuous reactor: A promising technology in colored wastewater treatment. Chem. Eng. J..

[13] Zhang H., Li F., Jia Q. (2009). Preparation of titanium nitride ultrafine powders by sol–gel and microwave carbothermal reduction nitridation methods. Ceram. Int..

[14] Yousefi E., Ghorbani M., Dolati A., Yashiro H. (2016). Facile synthesis of titanium nitride-graphene nanocomposite and its improved rate-dependent electroactivity with respect to lithium storage. Mater. Res. Bull..

[15] Ru J., Hua Y., Xu C., Zhang Q., Wang D., Gong K. (2014). Synthesis of TiN from FeTiO_3_ by microwave-assisted carbothermic reduction–nitridation. J. Alloys Compd..

[16] Gou H.P., Zhang G.H., Chou K.C. (2017). Phase evolution and reaction mechanism during reduction–nitridation process of titanium dioxide with ammonia. J. Mater. Sci..

[17] Tripathi M.K., Singh V.B., Singh H.K. (2015). Structure and properties of electrodeposited functional Ni–Fe/TiN nanocomposite coatings. Surf. Coat. Technol..

[18] Liu G., Pan Z., Zhang B., Xiao J., Xia G., Zhao Q., Shi S., Hu G., Xiao C., Wei Z. (2017). A novel TiN coated CNTs nanocomposite CNTs@TiN supported Pt electrocatalyst with enhanced catalytic activity and durability for methanol oxidation reaction. Int. J. Hydrogen Energy.

[19] Mani S.P., Srinivasan A., Rajendran N. (2015). Effect of nitrides on the corrosion behaviour of 316L SS bipolar plates for Proton Exchange Membrane Fuel Cell (PEMFC). Int. J. Hydrogen Energy.

[20] Su J., Lu N., Zhao J., Yu H., Huang H., Dong X., Quan X. (2012). Nano-cubic structured titanium nitride particle films as cathodes for the effective electrocatalytic debromination of BDE-47. J. Hazard. Mater..

[21] Jin Z., Li P., Xiao D. (2014). Enhanced Electrocatalytic Performance for Oxygen Reduction via Active Interfaces of Layer-By-Layered Titanium Nitride/Titanium Carbonitride Structures. Sci. Rep..

[22] Karthik T., Rathinamoorthy R., Murugan R. (2011). Enhancement of wrinkle recovery angle of cotton fabric using citric acid cross-linking agent with nano-TiO_2_ as a co-catalyst. Ind. Text..

[23] Ma J., Wu M., Du Y., Chen S., Li G., Hu J. (2009). Synthesis of nanocrystalline titanium nitride at low temperature and its thermal stability. J. Alloys Compd..

[24] Mousset E., Wang Z., Hammaker J., Lefebvre O. (2016). Physico-chemical properties of pristine graphene and its performance as electrode material for electro-Fenton treatment of wastewater. Electrochim. Acta.

[25] Bard A.J., Faulkner L.R. (2001). Fundamentals and applications. Electrochemical Methods, 2nd ed..

[26] Grewal Y.S., Shiddiky M.J., Gray S.A., Weigel K.M., Cangelosi G.A., Trau M. (2013). Label-free electrochemical detection of an Entamoeba histolytica antigen using cell-free yeast-scFv probes. Chem. Commun..

[27] Matthes B., Broszeit E., Aromaa J., Ronkainen H., Hannula S.P., Leyland A., Matthew A. (1991). Corrosion performance of some titanium-based hard coatings. Surf. Coat. Technol..

[28] Zhong Y., Xia X., Shi F., Zhan J., Tu J., Fan H.J. (2016). Transition Metal Carbides and Nitrides in Energy Storage and Conversion. Adv. Sci..

[29] Wetchakun N., Phanichphant S. (2008). Effect of temperature on the degree of anatase–rutile transformation in titanium dioxide nanoparticles synthesized by the modified sol–gel method. Curr. Appl. Phys..

[30] Dolat D., Moszyński D., Guskos N., Ohtani B., Morawski A.W. (2013). Preparation of photoactive nitrogen-doped rutile. Appl. Surf. Sci..

[31] Dolat D., Ohtani B., Mozia S., Moszyński D., Guskos N., Bieluń Z.L., Morawski A.W. (2014). Preparation, characterization and charge transfer studies of nickel—Modified and nickel, nitrogen co-modified rutile titanium dioxide for photocatalytic application. Chem. Eng. J..

[32] Bauer D.A., Herranen M., Ljungcrantz H., Carlsson J.O., Sundgren J.E. (1997). Corrosion behaviour of monocrystalline titanium nitride. Surf. Coat. Technol..

[33] Wang H., Zhang R., Yuan Z., Shu X., Liu E., Han Z. (2015). A comparative study of the corrosion performance of titanium (Ti), titanium nitride (TiN), titanium dioxide (TiO_2_) and nitrogen-doped titanium oxides (N–TiO_2_), as coatings for biomedical applications. Ceram. Int..

[34] Valdez B., Kiyota S., Stoytcheva M., Zlatev R., Bastidas J.M. (2014). Cerium-based conversion coatings to improve the corrosion resistance of aluminium alloy 6061-T6. Corros. Sci..

[35] Yang K.S., Mul G., Moulijn J.A. (2007). Electrochemical generation of hydrogen peroxide using surface area-enhanced Ti-mesh electrodes. Electrochim. Acta.

[36] Wang Y., Liu Y., Liu T., Song S., Gui X., Liu H., Tsiakaras P. (2014). Dimethyl phthalate degradation at novel and efficient electro-Fenton cathode. Appl. Catal. B Environ..

[37] Sirés I., Garrido J.A., Rodríguez R.M., Brillas E., Oturan N., Oturan M.A. (2007). Catalytic behavior of the Fe^3+^/Fe^2+^ system in the electro-Fenton degradation of the antimicrobial chlorophene. Appl. Catal. B Environ..

